# λ-Density Functional Valence Bond: A Valence Bond-Based Multiconfigurational Density Functional Theory With a Single Variable Hybrid Parameter

**DOI:** 10.3389/fchem.2019.00225

**Published:** 2019-04-16

**Authors:** Fuming Ying, Chen Zhou, Peikun Zheng, Jiamin Luan, Peifeng Su, Wei Wu

**Affiliations:** ^1^Fujian Provincial Key Laboratory of Theoretical and Computational Chemistry, Xiamen University, Xiamen, China; ^2^The State Key Laboratory of Physical Chemistry of Solid Surfaces, Xiamen University, Xiamen, China; ^3^College of Chemistry and Chemical Engineering, Xiamen University, Xiamen, China

**Keywords:** valence bond (VB) method, multi-configuration, density functional theory, multi-reference character, strong correlation

## Abstract

A new valence bond (VB)-based multireference density functional theory (MRDFT) method, named λ-DFVB, is presented in this paper. The method follows the idea of the hybrid multireference density functional method theory proposed by Sharkas et al. (2012). λ-DFVB combines the valence bond self-consistent field (VBSCF) method with Kohn–Sham density functional theory (KS-DFT) by decomposing the electron–electron interactions with a hybrid parameter λ. Different from the Toulouse's scheme, the hybrid parameter λ in λ-DFVB is variable, defined as a function of a multireference character of a molecular system. Furthermore, the *E*_C_ correlation energy of a leading determinant is introduced to ensure size consistency at the dissociation limit. Satisfactory results of test calculations, including potential energy surfaces, bond dissociation energies, reaction barriers, and singlet–triplet energy gaps, show the potential capability of λ-DFVB for molecular systems with strong correlation.

## Introduction

One of the major interests in quantum chemistry is the methodology development for electronic correlation energy calculation with an affordable computational cost. The basic multiconfigurational wave function methods, the multiconfigurational self-consistent field (MCSCF) method (Roos et al., 1980; Siegbahn et al., 1980), and the valence bond analog, valence bond self-consistent field (VBSCF) method (van Lenthe and Balint-Kurti, 1980, 1983), mainly consider static electron correlation, which is not covered in a single configuration-based wave function. Based on multiconfigurational wave function, the perturbative theory (PT), coupled-cluster (CC), or configuration interaction (CI) can be employed to cover dynamic correlation. With these post-self-consistent field (SCF) techniques, many high-level multiconfigurational wave function methods are proposed, including the multireference perturbation theory complete active space second-order perturbation theory (CASPT2) (Andersson et al., 1992), multireference second-order Møller-Plesset perturbation theory (MRMP2) (Nakano, 1993), valence bond second-order perturbation theory (VBPT2) (Wu et al., 1998; Chen et al., 2009), multireference configuration interaction (MRCI) (Siegbahn et al., 1981), valence bond configuration interaction (VBCI) (Wu et al., 2002; Song et al., 2004), breathing-orbital valence bond (BOVB) (Hiberty et al., 1992, 1994), and so on. The computational costs of these post-SCF multiconfigurational wave function methods increase rapidly with the size of active space.

Meanwhile, owing to its high efficiency for dynamic correlation calculation, the Kohn–Sham density functional theory (KS-DFT) is the most widely used electronic structure method (Hohenberg and Kohn, 1964; Kohn and Sham, 1965; Parr and Yang, 1989; Koch and Holthausen, 2001). Thus, the development of the multireference wave function-based DFT (MRDFT) method, in which the dynamic correlation is considered by DFT functionals and the static correlation is covered by a multiconfigurational wave function method, is promising because of its economical computational cost. Since the 1990s, many MRDFT schemes have been proposed (Lie and Clementi, 1974; Miehlich and Stoll, 1997; Filatov and Shaik, 1999; Gräfenstein and Cremer, 2000; Grafenstein and Cremer, 2005; Head-Gordon, 2003; Gusarov et al., 2004; Pérez-Jiménez et al., 2004; Yamanaka et al., 2006; Wu et al., 2007; Cembran et al., 2009; Kurzweil et al., 2009; Rapacioli et al., 2010; Sharkas et al., 2012; Ying et al., 2012; Manni et al., 2014; Zhou et al., 2017), most of which use the MCSCF/ complete active space self-consistent field (CASSCF) as the multireference wave function. Recently, two valence bond wave function-based MRDFT (DFVB) methods are presented: the first one is the dynamic correlation-corrected density functional valence bond (dc-DFVB) method (Ying et al., 2012), and the second one is the Hamiltonian matrix correction-based density functional valence bond (hc-DFVB) method (Zhou et al., 2017). These two methods are capable of providing satisfactory accuracy with relatively cheaper computational costs, compared to the currently existing post-VBSCF methods. However, these two methods still suffer from double counting error (DCE).

DCE is one of the key issues for MRDFT, because it is impossible to separate the static and dynamic correlations exactly. The range-separated scheme is considered to be helpful for MRDFT to avoid double counting error (Fromager et al., 2007, 2009). In the range-separated MRDFT, electron–electron interaction operator is decomposed into two components: the long-range term considered by the wave function method and the short-range term described by a density functional approximation. Recently, a multiconfigurational hybrid density functional theory, which is called multiconfigurational one-parameter hybrid (MC1H) approximation, is proposed by Sharkas et al. (2012). MC1H is based on a linear decomposition of electron–electron interactions with a hybrid parameter λ. It is shown that the accuracy of this multiconfigurational hybrid scheme matches that of the range-separated multiconfigurational hybrid method (Sharkas et al., 2012).

To remove the DCE from the DFVB methods, this paper presents a new VB-based MRDFT method, named λ-DFVB, by utilizing the MC1H scheme. Different from the MC1H scheme, which suggests setting λ as 0.25, the value of λ is variable in λ-DFVB, defined as a function of multireference character. Furthermore, the energy expression is modified to consider the dynamic correlation energies of dissociated fragments/atoms, ensuring the size consistency at the dissociation limit.

## Methodology

In VB theory, the many-electron wave function Ψ is expressed as a linear combination of VB functions (Hiberty and Shaik, 2008; Wu et al., 2011; Su and Wu, 2013),

(1) $$\begin{array}{l} \text{Ψ}=\underset{K}{\sum }C_{K}\Phi_{K}, \end{array}$$

where Φ_*K*_ and *C*_*K*_ are VB functions corresponding to a specific structure and its coefficient, respectively.

In spin-free quantum chemistry, VB function, which is an eigenfunction of spin and antisymmetric with respect to permutations of electron indices, is of the form

(2) $$\begin{array}{l} \Phi_{K}=Â\Omega_{0}\Theta_{K}, \end{array}$$

where Â is an antisymmetrizer for electron indices, Ω_0_ is an orbital product,

(3) $$\begin{array}{l} \Omega_{0}=\phi_{1}\left(1\right)\phi_{2}\left(2\right)\cdots \phi_{N}\left(N\right), \end{array}$$

and Θ_*K*_ is a spin eigenfunction (Pauncz, 1979), defined as

(4) $$\begin{array}{l} \Theta_{K}=2^{-1/2}\left[\alpha \left(k_{1}\right)\beta \left(k_{2}\right)-\beta \left(k_{1}\right)\alpha \left(k_{2}\right)\right]\\ \;\times 2^{-1/2}\left[\alpha \left(k_{3}\right)\beta \left(k_{4}\right)-\beta \left(k_{3}\right)\alpha \left(k_{4}\right)\right]\cdots \alpha \left(k_{p}\right)\cdots \alpha \left(k_{N}\right) \end{array}$$

In Equation 4, spin pairs (*k*_1_*, k*_2_), (*k*_−3_*, k*_4_), etc., correspond to covalent bonds in structure *K*, and *k*_*p*_ is for unpaired electrons.

Coefficients {*C*_*K*_} in Equation 1 can be obtained by solving the secular equation:

(5) $$\begin{array}{l} \text{H}\text{C}=E\text{M}\text{C}, \end{array}$$

where **H**, **M**, and **C** are Hamiltonian, overlap, and coefficient matrices, respectively.

In a similar fashion to molecular orbital methods, there are various *ab initio* classical VB methods (van Lenthe and Balint-Kurti, 1980, 1983; Hiberty et al., 1992, 1994; Hiberty and Shaik, 2002; Wu et al., 2002; Song et al., 2004; Chen et al., 2009). Among them, the valence bond self-consistent field (VBSCF) method is the basic one. In VBSCF, both VB structure coefficients {*C*_*K*_} and VB orbitals {φ_*i*_} are optimized simultaneously to minimize the total energy *E*. VB orbitals are usually expanded as linear combinations of basis functions.

(6) $$\begin{array}{l} \begin{array}{l} \phi_{i}=\underset{\mu }{\sum^{\;}}T_{\mu i}\chi_{\mu }. \end{array} \end{array}$$

VB orbitals may be taken as strictly localized hybrid atomic orbitals (HAOs), semilocalized bond-distorted orbitals (BDOs) (Mo et al., 1994, 1996), or delocalized overlap-enhanced orbitals (OEOs) (Bobrowicz and Goddard, 1977; Cooper et al., 1991), according to the specific purpose of a study. Analogous to the MCSCF/CASSCF method, VBSCF mainly considers static correlation.

VB structural weights can be evaluated by the Coulson–Chirgwin formula (Chirgwin and Coulson, 1950), which is an equivalence of the Mulliken population analysis,

(7) $$\begin{array}{l} W_{K}^{\;}=\underset{L}{\sum }C_{K}M_{KL}^{\;}C_{L}. \end{array}$$

VB structural weights are typically used to compare the relative importance of individual VB structures and can be helpful in the understanding of the correlation between molecular structure and reactivity.

In dynamic correlation-corrected density functional valence bond (dc-DFVB), the total energy can be expressed as

(8) $$\begin{array}{l} E^{\text{dc}-\text{DFVB}}\left[\rho \right]=\underset{\text{Ψ}}{\min}\left\{\langle \text{Ψ}|T+V_{\text{ext}}+W_{\text{ee}}|\text{Ψ}\rangle +E_{\text{C}}\left[\rho \right]\right\}, \end{array}$$

where *E*_C_[ρ] is obtained from a pure correlation functional with electronic density computed from the VBSCF wave function. The dc-DFVB method improves the VBSCF results, but it suffers from the double counting error due to the fact that it simply includes the total *E*_C_ energy in the total energy.

In the MC1H approximation by Sharkas et al. (2012), the energy of the MRDFT can be determined by minimizing the following expression:

(9) $$\begin{array}{l} E^{\text{MC1H}}=\underset{\text{Ψ}}{\min}\left\{\langle \text{Ψ}|T+V_{\text{ext}}+\lambda W_{\text{ee}}|\text{Ψ}\rangle +E_{\text{HXC}}^{\;\lambda \;}\left[\rho \right]\right\}, \end{array}$$

where *T, V*_ext_, and *W*_ee_ are the kinetic energy, external potential, and electron–electron interaction operators, respectively; $E_{\text{HXC}}^{\;\lambda \;}\left[\rho \right]$is the complement λ-dependent Hartree–exchange–correlation density functional for electronic density ρ, and λ is a coupling parameter. $E_{\text{HXC}}^{\;\lambda \;}\left[\rho \right]$is defined as (Sharkas et al., 2012):

(10) $$\begin{array}{l} E_{\text{HXC}}^{\;\lambda \;}\left[\rho \right]=\left(\text{1-}\lambda \right)\left(E_{\text{H}}\left[\rho \right]+E_{\text{X}}\left[\rho \right]\right)+\left(1-\lambda^{2}\right)E_{\text{C}}\left[\rho \right], \end{array}$$

where *E*_X_[ρ] and *E*_C_[ρ] are the exchange and correlation functionals, respectively. *E*_H_ is the Hartree energy, given as

(11) $$\begin{array}{l} E_{\text{H}}=\frac{1}{2}\iint \frac{\rho \left(r\right)\rho \left(r^{\prime }\right)}{\left|r-r^{\prime }\right|}drdr^{\prime }. \end{array}$$

Equation 10 turns to the KS-DFT formula when λ = 0.0, while it becomes wave function theory (WFT) if λ = 1.0. As suggested by Toulouse, the value of λ is approximately taken as 0.25.

It is clear that the parameter λ indicates the hybrid extent of the WFT and the KS-DFT. Based on the fact that multiconfiguration-based WFT is suitable for molecules with multireference character, while KS-DFT is a good tool for molecules with single-reference character, it is more reasonable to allow the value of λ to be different with different molecules. In this paper, we use a variable parameter for λ, which represents the multireference character of a molecule instead of a fixed value. There have been various indices for estimating multireference character or static correlation character (Zeische et al., 1997; Janssen and Nielsen, 1998; Leininger et al., 2000; Zanardi, 2002; Huang et al., 2006; Sears and Sherrill, 2008a,b; Tishchenko et al., 2008; Ramos-Cordoba et al., 2016; Benavides-Riveros et al., 2017; Ramos-Cordoba and Matito, 2017; Rodriguez-Mayorga et al., 2017), A large diagnostic value indicates a strong multireference character. These diagnostics include the T1 and D1 diagnostics in coupled-cluster wave functions (Lee and Taylor, 1989; Janssen and Nielsen, 1998; Leininger et al., 2000), the M diagnostic (Tishchenko et al., 2008), the 1-${\text{C}}_{0}^{2}$ diagnostic in CASSCF wave function (Sears and Sherrill, 2008a,b), the S_2_ diagnostic (Zeische et al., 1997; Zanardi, 2002; Huang et al., 2006); and the I_ND_ diagnostic (Ramos-Cordoba et al., 2016; Ramos-Cordoba and Matito, 2017), etc. In this paper, the concept of free valence is utilized to diagnose the multireference character of a molecule and is further used to determine the value of λ.

The free valence of an atom *A, F*_*A*_, is defined as

(12) $$\begin{array}{l} F_{A}=V_{A}-\underset{B,B\neq A}{\sum }O_{AB}, \end{array}$$

where *V*_*A*_ and *O*_*AB*_ are the total valence of atom *A* and the bond order between atoms *A* and *B*, respectively, defined as (Mayer, 2003)

(13) $$\begin{array}{l} V_{A}=\underset{\mu \in A}{\sum }2{\left(\text{D}\text{S}\right)}_{\mu \mu }-\underset{\mu ,\nu \in A}{\sum }{\left(\text{D}\text{S}\right)}_{\mu \nu }{\left(\text{D}\text{S}\right)}_{\nu \mu }, \end{array}$$

and

(14) $$\begin{array}{l} O_{AB}=\underset{\mu \in A}{\sum }\underset{\nu \in B}{\sum }\left[{\left(\text{D}\text{S}\right)}_{\mu \nu }{\left(\text{D}\text{S}\right)}_{\nu \mu }+{\left({\text{P}}^{s}\text{S}\right)}_{\mu \nu }{\left({\text{P}}^{s}\text{S}\right)}_{\nu \mu }\right], \end{array}$$

In Equations 13 and 14, **S** is the overlap matrix in terms of basis functions, D = P^α^ + P^β^ is the total density matrix, and P^*s*^ = P^α^ − P^β^ is the spin polarization density matrix for basis functions, where P^α^ and P^β^ are α and β density matrices, respectively.

The molecular free valence index *K* is defined as

(15) $$\begin{array}{l} K=\frac{\underset{A}{\sum }F_{A}^{\;}}{\underset{A}{\sum }V_{A}^{\;}}. \end{array}$$

It is clear that *K* ranges from 0 to 1. The value of *K* is small at the equilibrium geometry because all the atoms are bonded. For example, at the equilibrium distance, the Mayer bond order of H_2_ is 0.953 at VBSCF/cc-pVTZ. Then, *F*_H1_ = *V*_H1_-0.953 = 0.047 (subscript H1 denotes the first hydrogen atom); *K* = (0.047 + 0.047)/(1.0 + 1.0) = 0.047. At the dissociation limit, *K* = 1.0, as *O*_*AB*_ = 0 and *F*_*A*_ = *V*_*A*_.

Table S1 shows the comparisons of various diagnostics for diatomic molecules H_2_, HF, F_2_, N_2_, C_2_, and Cr_2_ in their equilibrium geometries, respectively. A large diagnostic value indicates a strong multireference character. Although the various diagnostic values are much different, their trends are in good agreement, showing the validation of *K*.

In this paper, the hybrid parameter λ is expressed as a function of the free valence index *K*. Based on some numerical investigations, shown in the Supporting Information, the function is defined as

(16) $$\begin{array}{l} \;\lambda \;=K^{\text{1/4}}. \end{array}$$

Clearly, the λ also ranges from 0.0 to 1.0, which satisfies the requirement of the hybrid parameter.

In Equation 10, the factor (1–λ^2^) of *E*_C_[ρ] arises from the fact that WFT covers the λ fraction of correlation and thus should be deducted from the functional *E*_C_[ρ]. In λ-DFVB, VBSCF wave function is used for the WFT part, and thus, only the static correlation of valence electrons, which results from the use of multideterminants, is covered by WFT. Therefore, the corresponding *E*_C_ functional should be approximately expressed as *E*_C_[ρ]–*E*_C_[ρ^LD^], where *E*_C_[ρ^LD^] is the *E*_C_ correlation energy determined by the electronic density of the leading determinant (LD), which is a single determinant with the largest coefficient in the VBSCF wave function. At a short distance, if dynamic correlation energy is dominating, *E*_C_[ρ]–*E*_C_[ρ^LD^] is small. At a long distance, the difference can be large because static correlation is largest at the bond dissociation limit. At last, the λ-DFVB energy is expressed as

(17) $$\begin{array}{l} E^{\;\lambda \text{-DFVB}}=\underset{\text{Ψ}}{\min}\left\{\langle \text{Ψ}|T+V_{\text{ext}}+\lambda W_{\text{ee}}|\text{Ψ}\rangle +E_{\text{HXC}}^{\;\lambda \text{-DFVB}}\left[\rho \right]\right\}, \end{array}$$

where

(18) $$\begin{array}{l} E_{\text{HXC}}^{\;\lambda \text{-DFVB}}\left[\rho \right]=\left(\text{1-}\lambda \right)\left(E_{\text{H}}\left[\rho \right]+E_{\text{X}}\left[\rho \right]\right)+E_{\text{C}}^{\;}\left[\rho \right]\\ \text{ -}\lambda^{2}\left(E_{\text{C}}^{\;}\left[\rho \right]-E_{\text{C}}^{\;}\left[\rho^{\text{LD}}\right]\right). \end{array}$$

When a molecule composed of two fragments/atoms A and B is fully dissociated, λ = 1.0. Then, the total energy can be expressed as

(19) $$\begin{array}{l} E_{\text{AB}}^{\;\lambda \;-\text{DFVB}}=E_{\text{AB}}^{\text{VBSCF}}+E_{\text{C}}\left[\rho^{\text{LD}}\right]. \end{array}$$

At the infinite distance, there is no overlap between fragments/atoms A and B; thus, the density of the leading determinant can be expressed as the sum of the densities (ρ_A_ + ρ_B_) of two fragments/atoms. As such, at the dissociation limit, *E*_C_[ρ^LD^] takes the dynamic correlations of dissociated fragments/atoms into account.

A λ-DFVB computation can be performed with the following steps:

1. Compute a VBSCF calculation to obtain the λ value and the VBSCF density ρ.
2. Compute $E_{\text{HXC}}^{\;\lambda \text{-DFVB}}\left[\rho \right]$ by Equation 18.
3. Compute the operator $v_{\text{HXC}}^{\;\lambda \text{-DFVB}}\left[\rho \right]$, which is defined as
  (20) $$\begin{array}{l} v_{\text{HXC}}^{\;\lambda \text{-DFVB}}\left[\rho \right]=\frac{\delta E_{\text{HXC}}^{\;\lambda \text{-DFVB}}\left[\rho \right]}{\delta \rho }. \end{array}$$
4. Optimize the λ-DFVB wave function with the following equation:
  (21) $$\begin{array}{l} \varepsilon_{\;}^{\;\lambda \text{-DFVB}}=\langle \text{Ψ}|T+V_{\text{ext}}+\;\lambda \;W_{\text{ee}}+\int drv_{\text{HXC}}^{\;\lambda \text{-DFVB}}\left[\rho \right]n\left(r\right)|\text{Ψ}\rangle . \end{array}$$
  where *n*(*r*) is the density operator, ρ(*r*) = 〈Ψ| *n*(*r*) |Ψ〉. The contribution of $v_{\text{HXC}}^{\;\lambda \text{-DFVB}}\left[\rho \right]$ is set into the VB Hamiltonian matrix. The computation is consistently iterative until convergence is achieved.
5. Obtain the λ-DFVB energy based on the wave function optimized in step 4:
  (22) $$\begin{array}{l} E_{\;}^{\;\lambda \text{-DFVB}}=\varepsilon_{\;}^{\;\lambda \text{-DFVB}}+E_{\text{HXC}}^{\;\lambda \;-\text{DFVB}}\left[\rho \right]\\ \;-\langle \text{Ψ}|\int drv_{\text{HXC}}^{\;\lambda \text{-DFVB}}\left[\rho \right]n\left(r\right)|\text{Ψ}\rangle . \end{array}$$

## Computational Details

The λ-DFVB method has been implemented in the Xiamen Valence Bond (XMVB) package (Su and Wu, 2013; Chen et al., 2015). All the VB calculations are performed by XMVB, while all KS-DFT calculations are carried out by General Atomic and Molecular Electron Structure System (GAMESS) (Schmidt et al., 1993; Gordon and Schmidt, 2005). Free valence and Mayer's bond order are computed with the VBSCF wave function. The MOLCAS 8.0 program (Aquilante et al., 2016) was used for CASSCF, MRCI, and CASPT2 calculations. The Davidson correction is considered for MRCI calculations. Two Generalized Gradient Approximation (GGA) functionals, Becke88 and Lee-Yang-Parr (BLYP) and Perdew-Wang 91 (PW91), are employed for the λ-DFVB calculations. The results of λ-DFVB with BLYP are shown in the main text, while those with PW91 are shown in the Supporting Information. In the dc-DFVB calculations, Lee-Yang-Parr (LYP) functional is used. For comparison, the corresponding results of B3LYP, BLYP, CASSCF, CASPT2, BOVB, and dc-DFVB are also provided.

Test calculations involve the potential energy surfaces of H_2_, HF, F_2_, N_2_, C_2_, and Cr_2_, the reaction barriers of the Diels–Alder (D-A) and Menshutkin reactions, and the energy gaps of carbon atom, oxygen atom, carbene (CH_2_), trimethylenemethane (TMM), and organometallics Fe(II)–porphyrin. The geometry of Fe(II)–porphyrin is optimized at the B3LYP level. The geometries of TMM, carbene, and the reactants and the transition states for the D-A reaction and the Menshutkin reaction are taken from previous papers (Ying et al., 2012; Huang et al., 2014; Zhou et al., 2017).

The cc-pVTZ (CCT) basis set was used for the potential energy surfaces of H_2_, HF, F_2_, N_2_, C_2_, and the energy gaps (except Fe(II)–porphyrin). 6-31G^*^ was used for the two chemical reactions. For Fe(II)–porphyrin, Lanl2DZ is for the iron atom and 6-31G^*^ is for the C, H, and N atoms. For Cr_2_, two basis sets, Stuttgart Royal Society of Chemistry (RSC) 1997 ECP (Andrae et al., 1990) and ANO-RCC-valence triple-zeta with polarization (VTZP) (Pou-Amérigo et al., 1995; Roos et al., 2005), were used.

## Results and Discussions

### The λ Values With Various Truncation Levels of VB Wave Function

**Figure 1:**
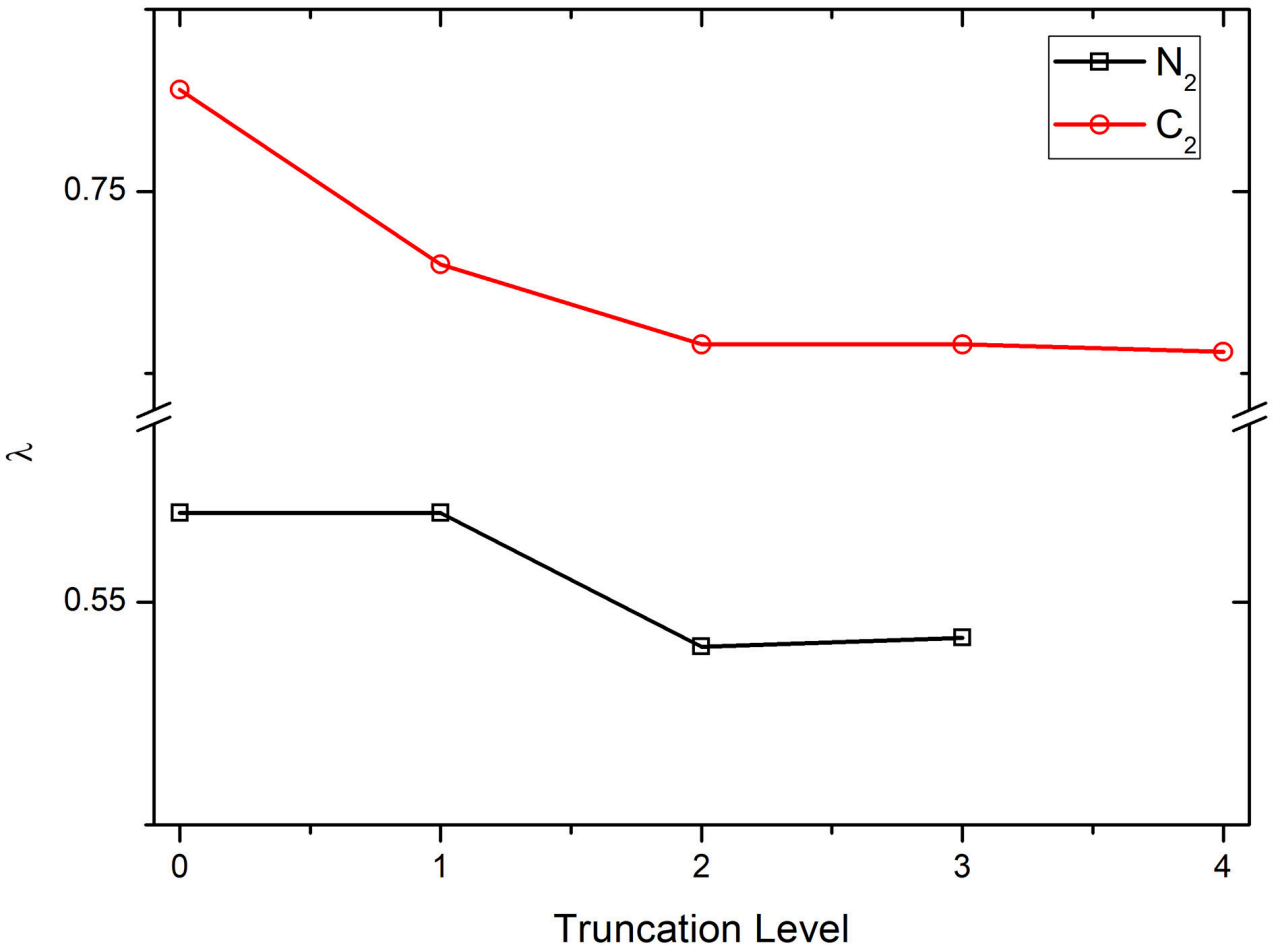
The λ values for N_2_ and C_2_ with various truncation levels of VB wave function.

First, the parameter λ is examined with various truncation levels of VBSCF wave function, including covalent structures only (denoted as COV, which is the 0th truncation level shown in Figure 1), covalent structures plus the 1st ~ *n*th order ionic structures, …, and all structures (denoted as CAS). Clearly, the CAS levels for N_2_ and C_2_ are the 3rd and 4th truncation levels, respectively. The numbers of VB structures in various truncation levels of N_2_ and C_2_ are listed in Table S2. Figure 1 displays the λ values of diatomic molecules N_2_ and C_2_ using OEOs, which are fully delocalized over the whole molecules, with the various truncation levels of VB wave function. As can be seen, the curves are almost flat, showing that the value of λ is not sensitive to the truncations of wave function. Meanwhile, including the ionic structures tends to reduce the λ value. In general, C_2_ has the larger λ value compared to N_2_ because C_2_ has the stronger multireference.

Table 1 displays the VBSCF and λ-DFVB energies of H_2_, F_2_, HF, N_2_, C_2_, and Cr_2_ with the CAS and COV levels of the VB wave function. The number of active electrons and active orbitals (*n*_e_, *n*_o_) are listed in the second column. The dynamic correlation correction of λ-DFVB, which is defined as the energy difference between VBSCF and λ-DFVB, is listed in the last column. The corresponding data for polyatomic molecules CH_3_-CH_3_, C_2_H_4_, and C_2_H_2_ are shown in Table S3. In general, the λ values of CAS are smaller than those of COV. The molecules with strong correlation tend to have the large λ values. Among them, H_2_ has the smallest dynamic correlation correction while Cr_2_ has the largest one.

**Table 1:** The λ-DFVB energies of H_2_, F_2_, HF, Cr_2_, N_2_, and C_2_ energies with variable λ values at their equilibrium geometries (a.u.).

	**Active space**		***E*^**VBSCF**^**	***E*^**λ−*DFVB***^**	**λ**	***E*^**corr**^*^a^***
N_2_	(6,6)	COV	−75.589398	−75.924588	0.764	−0.335190
		CAS	−75.637301	−75.952588	0.728	−0.315287
C_2_	(8,8)	COV	−109.065922	−109.524697	0.560	−0.458775
		CAS	−109.120064	−109.540330	0.546	−0.420266
H_2_	(2,2)	COV	−1.151417	−1.173454	0.465	−0.022037
		CAS	−1.151419	−1.172552	0.465	−0.021133
F_2_	(2,2)	COV	−198.828556	−199.505642	0.736	−0.677086
		CAS	−198.828556	−199.506120	0.727	−0.677564
HF	(2,2)	COV	−100.081614	−100.450733	0.437	−0.369119
		CAS	−100.081618	−100.450755	0.436	−0.369137
Cr_2_	(12,12)	COV	−172.514493	−173.471875	0.911	−0.957382
		CAS	−172.598336	−173.588538	0.809	−0.990202

a *E^corr^ = E^λ−DFVB^-E^VBSCF^*.

The λ-DFVB calculations in the next are carried out at the CAS level of the VB wave function.

### Potential Energy Surfaces of H_2_, HF, F_2_, N_2_, C_2_, and Cr_2_

**Figure 2:**
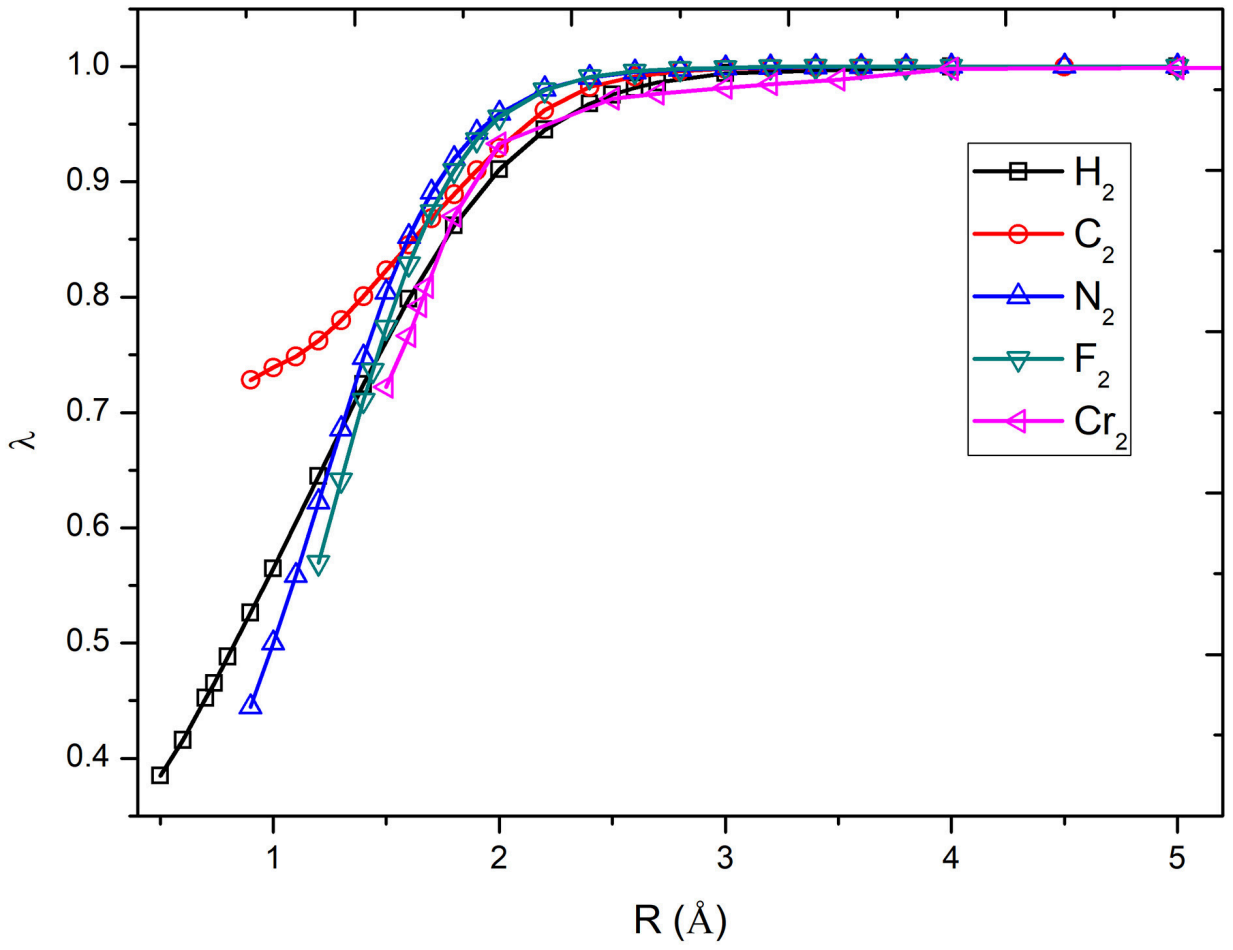
The curves of λ as functions of bond distances for diatomic molecules.

**Figure 3:**
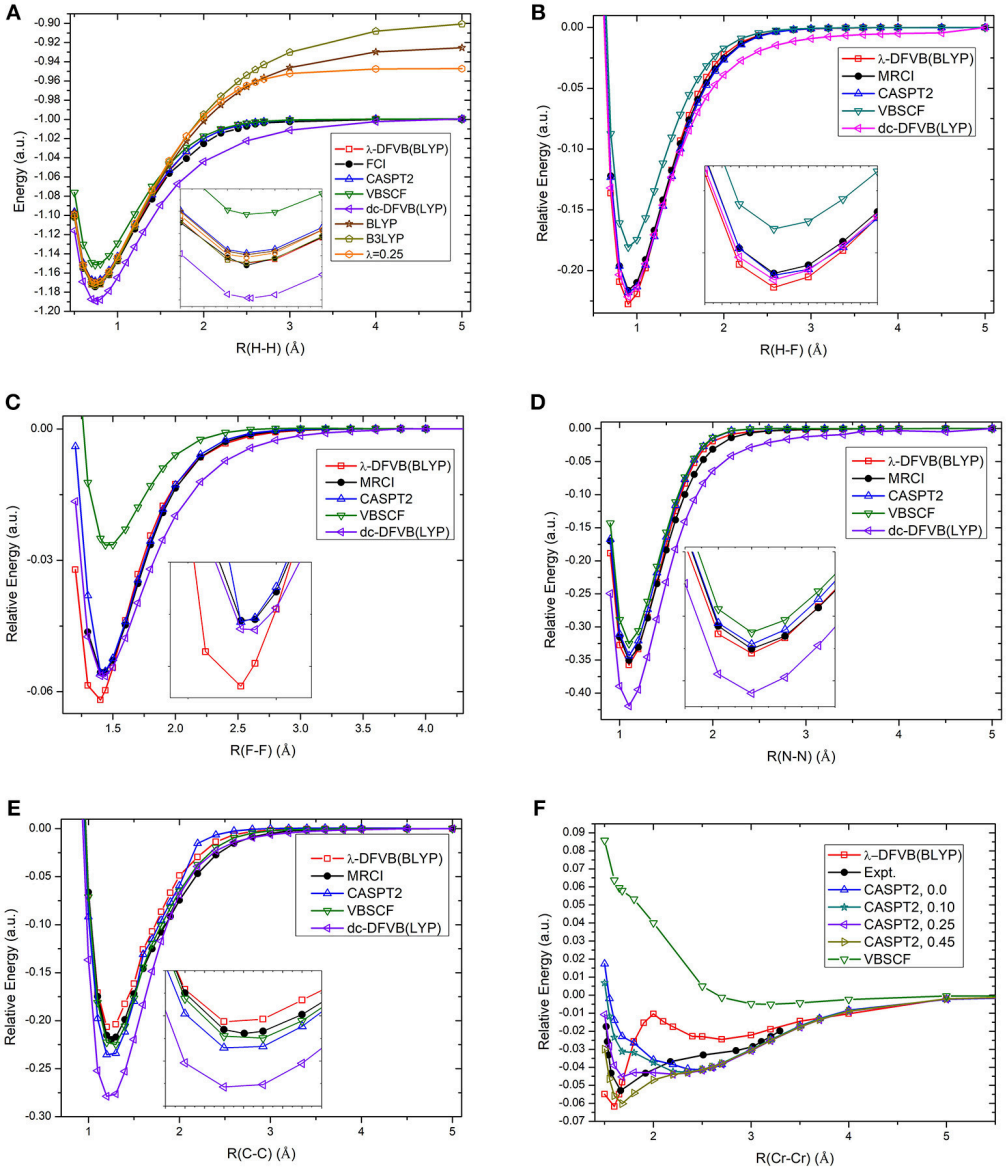
The PES curves of diatomic molecules with various methods: **(A)** H_2_; **(B)** HF; **(C)** F_2_; **(D)** N_2_; **(E)** C_2_; and **(F)** Cr_2_. The numbers in the brackets after “CASPT2” denote the IPEA shift.

The calculation of potential energy surface for bond breaking is one of the most rigorous tests for electronic structure methods. Figure 2 shows the curves of the λ values of diatomic molecules along the potential energy surfaces. As can be seen, the λ value goes up with the increase in bonding distance and reaches to 1.0 at the dissociation limit. For example, the λ value of H_2_ is 0.465 at the equilibrium bond distance (0.74 Å), while it is 1.0 at the dissociation limit. The other curves share similar behavior. It can be found that the molecules with strong correlation have large λ values in the short distances, showing the large portions of electron–electron interaction energy computed by the VB wave function methods. Based on the λ values shown in Figure 3, the bond dissociation curves of H_2_, HF, F_2_, N_2_, C_2_, and Cr_2_ by λ-DFVB are plotted in Figure 3. For comparison, the PES curves with various WFT and KS-DFT methods are also shown.

It can be seen from Figure 3A that the restricted B3LYP and BLYP calculations for the H_2_ molecule go to the wrong dissociation limits, as expected. Both VBSCF and dc-DFVB predict the dissociation correctly. However, VBSCF gives a higher energy at the equilibrium geometry due to the lack of dynamic correlation, while the dc-DFVB curve is underneath the full CI one because of the double counting error. Encouragingly, the performance of λ-DFVB is excellent, virtually overlapped with the full CI and CASPT2 ones along the whole curve. If one zooms in the figure, it can be found that the λ-DFVB curve is the closest to that of the full CI near the equilibrium geometry.

For the HF molecule, shown in Figure 3B, the λ-DFVB curve is quite close to those by dc-DFVB, MRCI, and CASPT2. Interestingly, although the dc-DFVB curve virtually overlaps with the others around the equilibrium geometry, it deviates from the others at about 1.7 Å and converges again at the dissociation limit. For the F_2_ results in Figure 3C, the VBSCF curve is the highest, while that of the dc-DFVB is the lowest after 1.60 Å. The λ-DFVB curve almost coincides with those of the MRCI and CASPT2 after 1.60 Å. However, the λ-DFVB curve is lowest around the equilibrium distance. For the N_2_ curves, shown in Figure 3D, the performance of λ-DFVB is very close to MRCI and CASPT2, better than those of VBSCF and dc-DFVB. For the C_2_ curves in Figure 3E, the three VB methods (VBSCF, dc-DFVB, and λ-DFVB) and CASPT2 predict the bonding dissociation of C_2_ in a similar fashion. It is shown that the λ-DFVB curve is slightly higher than that of the VBSCF around the equilibrium bond length, indicating that the dynamic correlation does not play a key role in the relative energy surface.

It is well-known that Cr_2_, which has sextuple bond with a small bonding energy, is a challenging molecule to quantum chemical methods due to its strong multireference character. Traditional KS-DFT calculations are unable to provide the potential energy surface properly (Brynda et al., 2009). For CASPT2, different values of the ionization potential and electron affinity (IPEA) shift lead to different results (Ruipierez et al., 2011). Figure S1 displays the CASSCF curves with the Stuttgart RSC 1997 ECP basis set (abbreviated as ECP) and the ANO-RCC-VTZP basis set (abbreviated as ANO), and the VBSCF curve with the ECP. It is found that the three curves are quite similar, and all of them predict a minimum around 3.0 Å, far away from the experimental equilibrium distance. In Figure 3F, ECP is used for VBSCF and λ-DFVB, and ANO is used for CASPT2. As expected, CASPT2 is sensitive to the value of the IPEA shift (Ruipierez et al., 2011; Manni et al., 2014). The CASPT2 curve with a value of 0.45 a.u. is close to that of the experimental. It is interesting that λ-DFVB successfully predicts the global minimum around 1.60 Å. Meanwhile, the barrier around 2.8 Å is in agreement with the curves computed by the modified generalized valence bond (MGVB) method and the curve by the MC-PDFT method (Manni et al., 2014).

### The Bond Dissociation Energies of Diatomic Molecules

The computed bond dissociation energies (BDEs; *D*_e_) for the six diatomic molecules at their optimized geometries are shown in Table 2. The BDEs of BLYP and B3LYP are computed as the energy difference between the equilibrium bond distances and the sum of atomic energies. Generally speaking, CASPT2 and MRCI perform well, showing the small deviations from the experimental data. With a large IPEA shift of 0.45 a.u., CASPT2 provides a satisfactory result for Cr_2_. Both BLYP and B3LYP are unable to provide the proper descriptions for C_2_ and Cr_2_ molecules, as mentioned in literature (Carlson et al., 2015; Kepp, 2017).

**Table 2:** The computed *D*_e_ for diatomic molecules (in kcal/mol).

	**CASPT2**	**MRCI**	**BLYP**	**B3LYP**	**VBSCF**	**BOVB**	**dc-DFVB**	**λ-DFVB**	**Expt**
H_2_	106.1	109.4	109.5	110.3	95.3	95.3	118.9	109.1	109.5 (Linstrom and Mallard, 2011; Johnson, 2018)
HF	133.8	135.8	139.4	138.0	113.4	124.4	139.0	142.9	141.3 (Linstrom and Mallard, 2011; Johnson, 2018)
F_2_	34.0	34.9	52.6	40.1	16.8	33.9	35.7	38.9	38.2 (Linstrom and Mallard, 2011; Johnson, 2018)
N_2_	215.6	219.9	242.3	230.2	204.1	238.6	263.2	224.3	228.5 (Linstrom and Mallard, 2011; Johnson, 2018)
C_2_	149.5	137.8	137.5	121.3	137.3	–	176.2	137.4	148.0 (Leininger et al., 1998)
Cr_2_	28.4(0.25)*^*a*^* 37.8(0.45)*^*a*^*	–	–	–	–	–	–	38.7	33.9 (Casey and Leopold, 1993)

a *Values in parentheses are the IPEA shifts used in CASPT2 calculations*.

Because of the lack of dynamic correlation, VBSCF is unable to provide satisfactory BDEs for H_2_, HF, F_2_, and N_2_. Analogous to CASSCF, VBSCF is unable to describe the Cr–Cr bonding properly, but it predicts the bonding of C_2_ quite well. BOVB uses different orbitals for different VB structures to consider the dynamic electron correlation. It cannot be employed in the molecules with large active spaces (for example, C_2_ and Cr_2_ in this work). In the BOVB calculations, the active orbitals are HAOs, while the remaining orbitals are OEOs. It is shown that the BOVB results are better than those of the VBSCF. By incorporating *E*_C_ functional into VBSCF, the BDEs of dc-DFVB are larger than their corresponding VBSCF values and are mostly overestimated for N_2_ and C_2_ compared to the experimental data, because of the DCE. Similar to VBSCF, dc-DFVB is also incapable of predicting the stable Cr–Cr bonding.

For λ-DFVB, in general, its computational results are excellent, close to the MRCI and CASPT2 values and superior to the BOVB and dc-DFVB ones. For N_2_, the λ-DFVB value is 224.3 kcal/mol, which is close to the experimental data of 228.5 kcal/mol and even better than the CASPT2 one. The λ-DFVB value of C_2_, 137.4 kcal/mol, is close to the MRCI result of 137.8 kcal/mol and that of ICMRCI+Q/cc-pVTZ, 138.8 kcal/mol (Pradhan et al., 1994). For Cr_2_, the BDE value of λ-DFVB is 38.7 kcal/mol, close to the experimental data, 33.9 kcal/mol, and the CASPT2 result of 32.8 kcal/mol with the IPEA shift of 0.45 a.u. and cc-pVTZ-DK basis set by Truhlar (Carlson et al., 2015), better than the MC-PDFT result, 13.8 kcal/mol, at the tPBE/cc-pVTZ-DK level (Manni et al., 2014).

### Chemical Reaction Barriers

Table 3 lists the reaction barriers for the Diels–Alder reaction and the Menshutkin reaction. The λ values for the reactants and transition states of the two reactions are shown in Table S4. It can be seen that VBSCF overestimates the values of barriers due to the lack of dynamic correlation, while KS-DFT underestimates the reaction barriers, as expected. The dc-DFVB results are much improved from those of the VBSCF. The performance of λ-DFVB is very good. For the Diels–Alder reaction, the barrier of 24.6 kcal/mol is very close to those of the CASPT2 (23.5 kcal/mol) and the experimental (23.3 kcal/mol). For the Menshutkin reaction, the λ-DFVB value of 32.2 kcal/mol is even better than that of CASPT2, 40.5 kcal/mol, compared to the experimental value of 33.0 kcal/mol.

**Table 3:** The barriers of the D-A and Menshutkin chemical reactions (in kcal/mol).

	**Menshutkin reaction**	**Diels–Alder reaction**
	H_3_N + CH_3_Cl → H_3_N…${\text{CH}}_{3}^{+}$…Cl^−^ → H_3_${\text{NCH}}_{3}^{+}$ + Cl^−^ Reactant TS Product	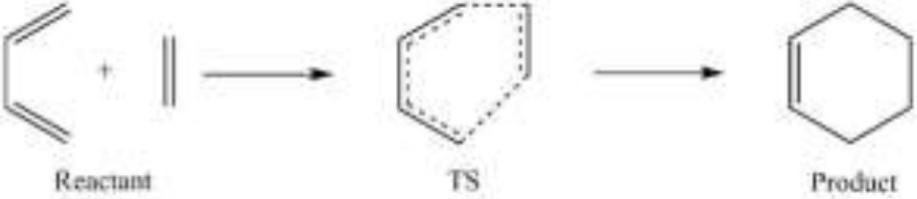
CASPT2	40.5	23.5
BLYP	27.0	17.7
B3LYP	29.9	21.3 (Zhou et al., 2017)
VBSCF	41.5	41.7
dc-DFVB	38.5	34.5
λ-DFVB	32.4	24.6
Expt	33.0 (Webb and Gordon, 1999)	23.3 ± 2 (Webb and Gordon, 1999; Guner et al., 2003)

### The Excitation Energy Gaps

The singlet–triplet energy gaps of carbon atom, oxygen atom, carbene (CH_2_), and trimethylenemethane (TMM) by various methods are shown in Table 4, the corresponding λ values for the λ-DFVB calculations are shown in Table S4. As expected, the KS-DFT results show very large deviations from the experimental data and the CASPT2 results for all atoms and molecules except porphyrin. Meanwhile, VBSCF predicts the excitation and transition energies quite well. Compared to VBSCF and dc-DFVB, the performance of λ-DFVB is much improved, particularly for molecules. As can be seen, the deviation values from the experimental data are 1.6 and 0.3 kcal/mol for CH_2_ and TMM, respectively, even smaller than those of CASPT2.

**Table 4:** The singlet–triplet energy gaps of C, O, carbene (CH_2_), and trimethylenemethane (TMM) (in kcal/mol).

		**VBSCF**	**CASPT2**	**BLYP**	**B3LYP**	**dc-DFVB**	**λ-DFVB**	**Expt**
**C**	^3^P → ^1^D	34.5	30.0	39.2	40.3	24.6	25.3	29.1 (Ess et al., 2011)
**O**	^3^P → ^1^D	50.3	46.3	60.9	62.4	40.3	41.1	45.4 (Ess et al., 2011)
**CH**_**2**_	^3^B_1_ → ^1^B_1_	39.2	26.9	7.3	29.8	25.9	31.3	32.9 (Ess et al., 2011)
**TMM**	^3^${\text{A}}_{2}^{{\;}^{\text{′}}}$→^1^A_1_	21.5	20.1	34.7	43.9	14.7	18.4	18.1 (Li and Paldus, 2008)

**Figure 4:**
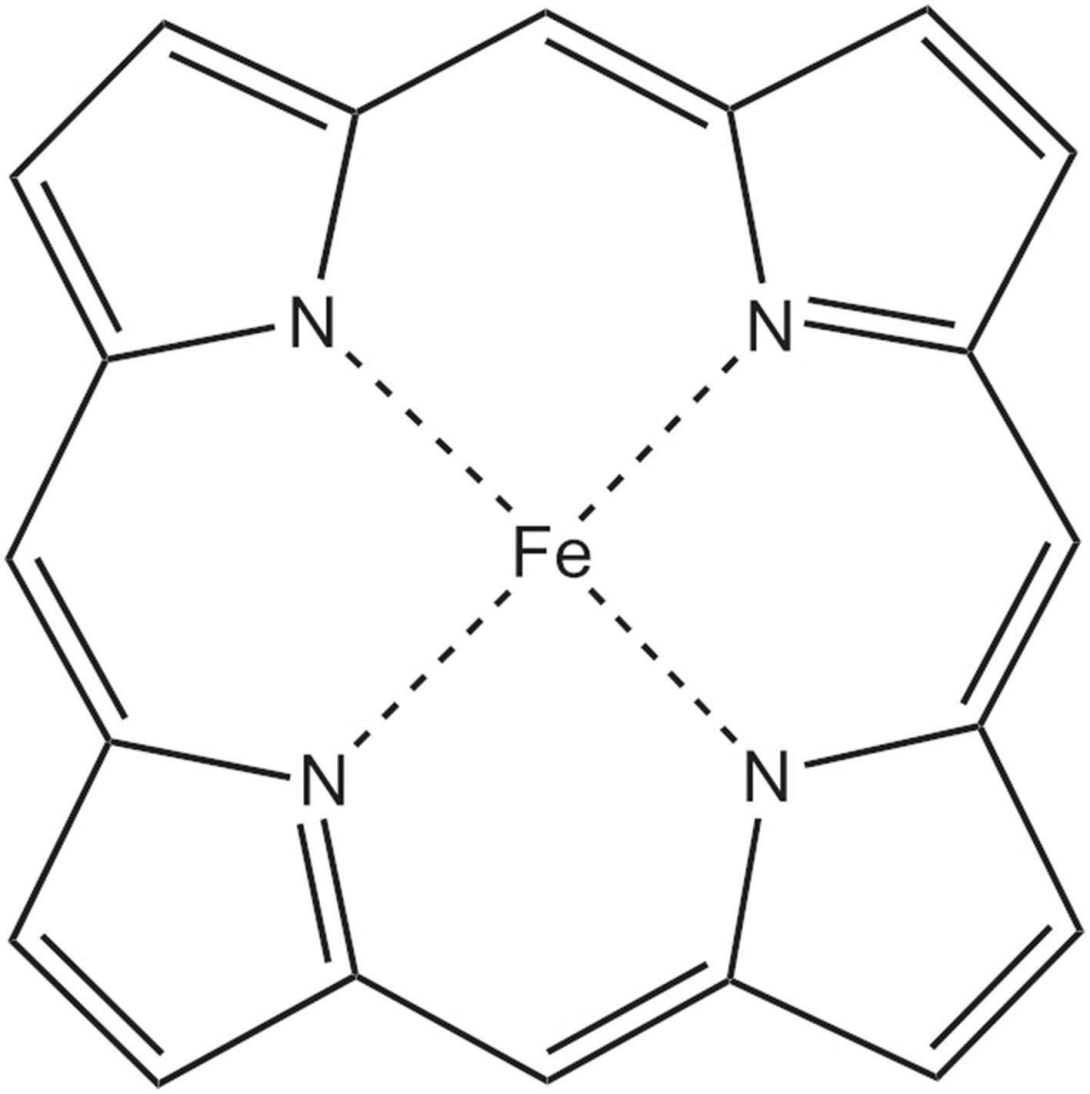
The Fe(II)–porphyrin complex.

Fe(II)–porphyrin, shown in Figure 4, is an important organometallic compound comprising the active center of several important biological proteins. Experimental studies show that the triplet state is lower than the quintet state. However, it is a challenge to describe the relative stability of the quintet and triplet states correctly with multireference wave function methods. Manni and Alavi (2018) and Smith et al. (2017) found that only the CASSCF calculations with very large active spaces are able to predict the triplet–quintet gaps properly. For the VB and CASSCF calculations, the active space includes the six valence electrons of the metal center and its five 3d orbitals. Our computed triplet–quintet gaps for Fe(II)–porphyrin with various methods are displayed in Table 5. It is found that the VBSCF, dc-DFVB, CASSCF, and CASPT2 results predict that the quintet state is more stable than the triplet state. Only λ-DFVB and B3LYP describe the gap correctly. The λ-DFVB result of 2.40 kcal/mol is close to the reference data of ca 3.0 kcal/mol by stochastic-CASSCF (32,34) (Manni and Alavi, 2018) and 2.0 kcal/mol by heat-bath configuration interaction with semistochastic perturbation theory at the active space (44,44) (Olivares-Amaya et al., 2015).

**Table 5:** The triplet–quintuplet energy gap of Fe(II)–porphyrin by various methods (in kcal/mol).

**Method**	**Energy gap**
VBSCF	−26.2
dc-DFVB	−17.0
λ-DFVB	2.4
MCSCF (6,5)	−26.0
CASPT2 (6,5)	−6.7
B3LYP (Kozlowski et al., 1998)	6.2
SHCI (44,44) (Smith et al., 2017)	1.9
Stoch-CAS (32,34) (Manni and Alavi, 2018)	3.1

The λ-DFVB with the PW91 functional is also performed for BDEs, chemical barriers, and singlet–triplet gaps. In general, the results are close to those of λ-DFVB with BLYP. Details are shown in Tables S5–S7. The structure weights and orbitals of N_2_ are displayed in Figures S2, S3 showing that the wave function of λ-DFVB is similar to that of the VBSCF.

## Conclusion

A new hybrid multireference density functional theory method based on the VB theory, named λ-DFVB, is presented in this paper. Based on the MC1H approximation presented by Sharkas et al. (2012), λ-DFVB combines VBSCF and KS-DFT with a linear decomposition for electron–electron interactions. In λ-DFVB, the hybrid parameter λ is variable, ranging from 0.0 to 1.0, and defined as a function of the free valence index *K*, which diagnoses the multireference character for a given system. Furthermore, an additional correlation term, *E*_C_(ρ^LD^), is introduced to consider the correlation energies of fragments/atoms in the dissociation limit, which ensures that the λ-DFVB method is size consistent.

The λ-DFVB method was carefully examined by performing test calculations for various chemical properties, including potential energy surfaces, bond dissociation energies, chemical reaction barriers, and singlet–triplet energy gaps. The performance of λ-DFVB is promising, close to those of CASPT2 and MRCI. Especially, the proper descriptions of the Cr_2_ bonding and the triplet–quintet gap of the model molecule Fe(II)–porphyrin in their equilibrium geometries, which are challenging both to the WFT and DFT methods, show the capability of λ-DFVB for strong correlation systems.

The λ-DFVB method shares its dual advantage. On the one hand, λ-DFVB improves the accuracy of the VBSCF method by incorporating dynamic correlation; on the other hand, it overcomes some problems with KS-DFT that result from the use of a single determinant. Though the current strategy of the λ-DFVB is applied to the GGA functionals in this paper, it will be extended to more general functionals, such as hybrid functionals and meta-GGA functionals, which will be discussed in the near future.

## Author Contributions

FY: the implementation of λ-DFVB and numerical tests; CZ: the implementation of λ-DFV and numerical tests; PZ and JL: numerical tests; PS and WW: methodology and formula derivations.

### Conflict of Interest Statement

The authors declare that the research was conducted in the absence of any commercial or financial relationships that could be construed as a potential conflict of interest.
