# Supplementary material for: λ-Density Functional Valence Bond: A Valence Bond-Based Multiconfigurational Density Functional Theory With a Single Variable Hybrid Parameter
Source: Front Chem. 2019 Apr 16;7:225. doi: 10.3389/fchem.2019.00225 (PMC6476929; doi:10.3389/fchem.2019.00225)

**λ-DFVB: A valence bond based multi-configurational density functional theory with a single variable** **hybrid parameter**

Fuming Ying, Chen Zhou, Peikun Zheng, Jiamin Luan, Peifeng Su, ^*^ Wei Wu ^*^

Fujian Provincial Key Laboratory of Theoretical and Computational Chemistry, The State Key Laboratory of Physical Chemistry of Solid Surfaces, and College of Chemistry and Chemical Engineering, Xiamen University, Xiamen, Fujian 361005, China.

Table S1. Comparison of the different diagnostics for diatomic molecules in their equilibrium distances.

|  | *V* | *F* | *K* | T1 | D1 | M | $\boldsymbol{1-}\boldsymbol{C}_{\boldsymbol{0}}^{\boldsymbol{2}}$ | I_ND_ | S_2_ |
| --- | --- | --- | --- | --- | --- | --- | --- | --- | --- |
| H­­_2_ | 1.005 | 0.047 | 0.047 | 0.006*^a^* | 0.008*^a^* | 0.024 | 0.012*^a^* | 0.024 | 0.028 |
| HF | 2.033 | 0.074 | 0.036 | 0.007*^b^* | 0.012*^b^* | 0.019 | / | 0.019 | 0.023 |
| C_2_ | 3.864 | 1.084 | 0.281 | 0.038*^a^* | 0.086*^a^* | 0.403*^a^* | 0.291*^a^* | 0.542 | 0.419 |
| N_2_ | 3.001 | 0.292 | 0.097 | 0.013*^a^* | 0.026*^a^* | 0.062*^a^* | 0.072*^a^* | 0.146 | 0.146 |
| F_2_ | 0.948 | 0.279 | 0.294 | 0.011*^a^* | 0.029*^a^* | 0.131 | 0.067*^a^* | 0.140 | 0.166 |
| Cr_2_ | 6.070 | 2.598 | 0.428 | 0.047*^c^* | 0.078*^c^* | 0.398 | 0.386*^c^* | 0.671 | 0.957 |

*a* Fogueri, U. R.; Kozuch, S.; Karton, A.; Martin, J. M. L. *Theor. Chem. Acc.* **2012**, *132*, 1291.

*b* Janssen, C. L.; Neilsen, I. M. B. *Chem. Phys. Lett.* **1998**, *290*, 423-430

*c* Jiang, W.; DeYonker, N.; Wilson, A. K. *J. Chem. Theor. Comput.* **2012**, *8*, 460-468.

Table S2. The number of VB structures in different levels.

| Truncation level | N_2_ | C_2_ |
| --- | --- | --- |
| 0 | 5 | 14 |
| 1 | 65 | 294 |
| 2 | 155 | 1134 |
| 3 | 175 | 1694 |
| 4 | - | 1764 |
| Total | 175 | 1764 |

Table S3. The VB energies and λ values with covalent and full structures at their equilibrium geometries (a.u.).

|  | Active space | VB set | *E*^VBSCF^ | *E*^λ-DFVB^ | λ | *E*^corr^ |
| --- | --- | --- | --- | --- | --- | --- |
| CH_3_-CH_3_ | (2,2) | COV | -79.275290 | -79.801140 | 0.262 | -0.525750 |
|  |  | CAS | -79.275290 | -79.801145 | 0.262 | -0.525855 |
| CH_2_=CH_2_ | (4,4) | COV | -78.104301 | -78.572534 | 0.424 | -0.468233 |
|  |  | CAS | -78.117004 | -78.576219 | 0.413 | -0.459215 |
| CH≡CH | (6,6) | COV | -76.912872 | -77.320758 | 0.497 | -0.407886 |
|  |  | CAS | -76.950891 | -77.336423 | 0.482 | -0.385532 |

Table S4. The λ values for polyatomic molecular systems.

|  |  | Active space | λ |
| --- | --- | --- | --- |
| D-A | Reactant | (4,3) | 0.422 |
|  | Transit state | (4,3) | 0.436 |
| Menshutkin | Reactant | (6,6) | 0.296 |
|  | Transit state | (6,6) | 0.273 |
| C | ^3^P | (4,4) | 1.000 |
|  | ^1^D | (4,4) | 1.000 |
| O | ^3^P | (3,4) | 1.000 |
|  | ^1^D | (3,4) | 1.000 |
| CH_2_ | ^3^B_1_ | (2,2) | 0.754 |
|  | ^1^B_1_ | (2,2) | 0.769 |
| TMM | ^3^A_2_^’^ | (4,4) | 0.553 |
|  | ^1^A_1_ | (4,4) | 0.568 |
| Fe(II)-porphyrin | ^5^A_1g_ | (6,5) | 0.440 |
|  | ^3^A_2g_ | (6,5) | 0.384 |

Table S5. The computed BDEs by λ-DFVB with the different values of λ.

|  |  | PW91 | λ-DFVB*^a^* | | | | | | | |  |
| --- | --- | --- | --- | --- | --- | --- | --- | --- | --- | --- | --- |
|  |  |  | λ=K^1/2^ | | λ=K^1/3^ | | λ=K^1/4^ | | λ=K^1/5^ | | Expt |
|  |  |  | BLYP | PW91 | BLYP | PW91 | BLYP | PW91 | BLYP | PW91 |  |
| H_2_ | (2,2) | 105.2 | 107.1 | 100.0 | 107.8 | 101.4 | 109.1 | 103.0 | 109.1 | 103.5 | 109.5 |
| HF | (2,2) | 141.2 | 149.2 | 137.2 | 145.4 | 135.4 | 142.9 | 134.4 | 141.2 | 133.9 | 141.3 |
| N_2_ | (6,6) | 244.6 | 226.1 | 208.2 | 224.6 | 210.3 | 224.3 | 212.5 | 224.3 | 214.6 | 228.5 |
| C­_2_ | (8,8) | 160.9 | 119.0 | 113.6 | 130.1 | 127.3 | 137.4 | 136.2 | 142.5 | 142.5 | 148.0 |
| F_2_ | (2,2) | 56.9 | 47.7 | 34.5 | 41.4 | 32.0 | 38.9 | 30.7 | 37.0 | 30.1 | 38.2 |
| Cr_2_ | (12,12) | 34.1 | 60.9 | 34.7 | 46.5 | 24.1 | 38.7 | 18.6 | 33.9 | 15.4 | 33.9 |

*a* All are obtained with full structure calculation.

Table S6. The barriers of the D-A and Menshutkin reactions by λ-DFVB with PW91.

|  | Active space | PW91 | dc-DFVB | λ-DFVB | Expt. |
| --- | --- | --- | --- | --- | --- |
| D-A | (6,6) | 10.8 | 21.3 | 24.6 | 23.3 ± 2 |
| Menshutkin | (4,3) | 25.5 | 39.2 | 33.0 | 33.0 |

Table S7. The vertical excitation energy of formaldehyde (CH_2_O) and the singlet-triplet gaps (in kcal/mol) of C, O, CH_2_ and TMM by λ-DFVB with PW91.

|  |  | PW91 | dc-DFVB | λ-DFVB | Expt |
| --- | --- | --- | --- | --- | --- |
| CH_2_O | *n*→*π** | 73.8 | 97.0 | 90.0 | 93.9 |
| C | ^3^P→^1^D | 43.0 | 27.8 | 29.2 | 29.1 |
| O | ^3^P→^1^D | 65.2 | 43.7 | 45.7 | 45.4 |
| CH_2_ | ^3^B_1_→^1^ B_1_ | 7.2 | 31.3 | 34.8 | 32.9 |
| TMM | ^3^A_2_^’^→^1^A_1_ | 36.5 | 17.7 | 18.1 | 18.1 |

Figure S1. Comparison of VBSCF with CASSCF with different basis sets of Cr_2_.


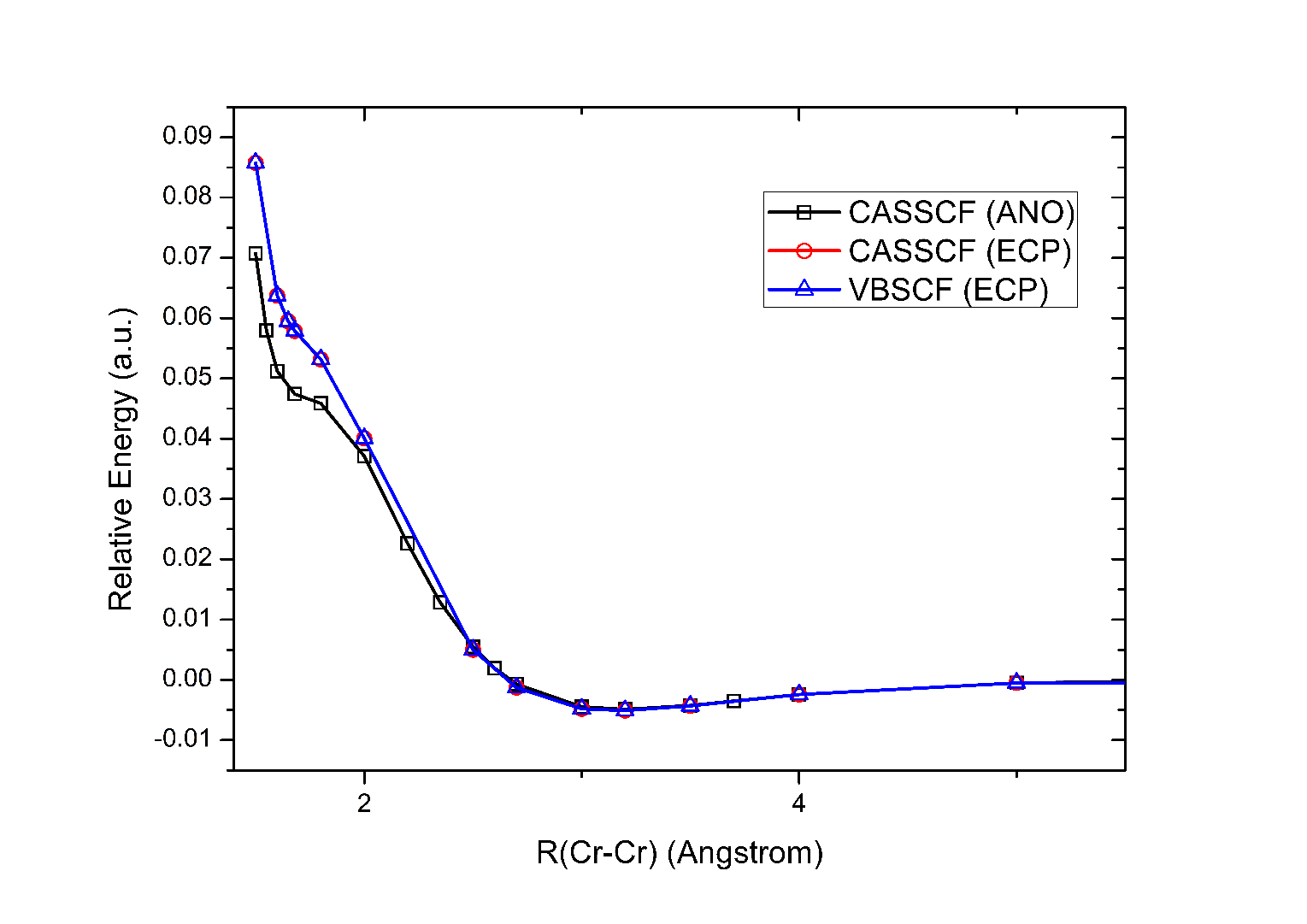


Figure S2. The curves of the weights of the important structures for N_2_ along the PES.*


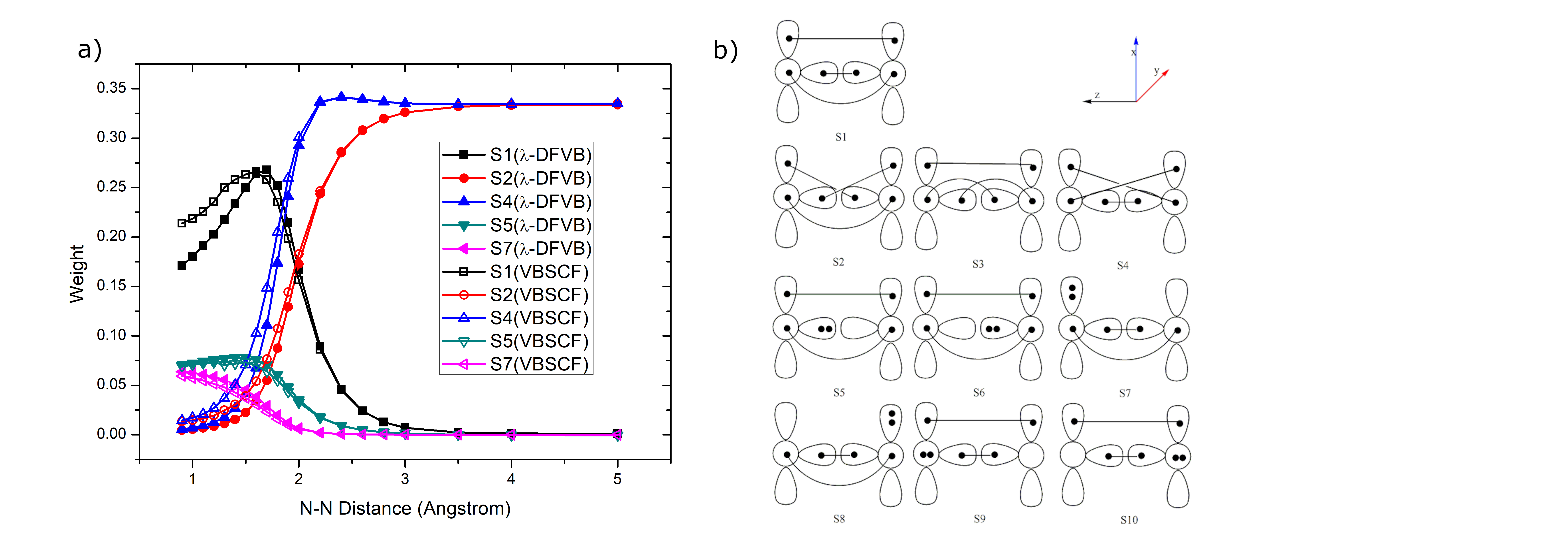


* Structures S2 and S3; S5 and S6; S7, S8, S9 and S10 are degenerate. As such only the weights of S1, S2, S4, S5 and S7 are shown.

Figure S3. The valence orbitals for N_2,_ optimized by VBSCF and λ-DFVB.


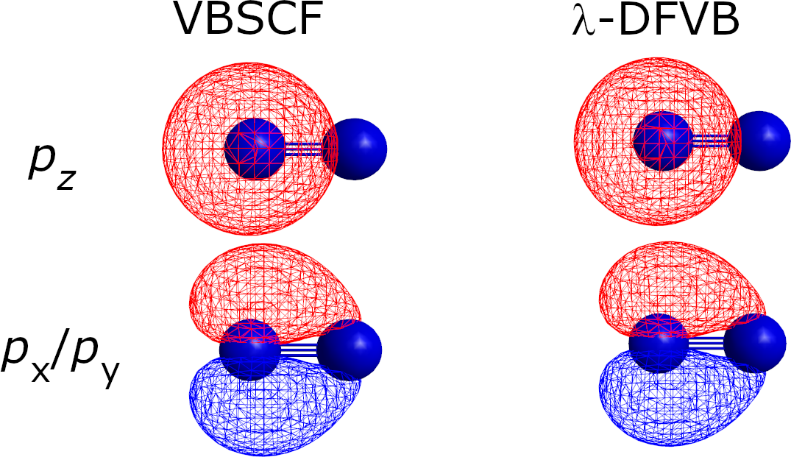

Supplement: Supplementary file 1 [file Table_1.DOCX]
